# multiDEGGs: Single or Multiomic Differential Network Analysis for Biomarker Discovery and Feature Engineering for Predictive Modeling

**DOI:** 10.34133/csbj.0001

**Published:** 2026-03-18

**Authors:** Elisabetta Sciacca, Susan S. Wang, Costantino Pitzalis, Myles J Lewis

**Affiliations:** ^1^ Department of Biomedical Sciences, Humanitas University, Milan, Italy.; ^2^ Centre for Experimental Medicine and Rheumatology, William Harvey Research Institute, Barts and The London School of Medicine and Dentistry, Queen Mary University of London, London, UK.; ^3^ Barts Health NHS Trust and National Institute for Health and Care Research (NIHR) Barts Biomedical Research Centre (BRC), London, UK.; ^4^ IRCCS Istituto Clinico Humanitas, Rozzano (MI), Italy.

## Abstract

**Motivation:** Modern clinical trials increasingly leverage high-throughput omic data for patient stratification and biomarker discovery. While traditional differential gene expression analysis disregards the networked nature of molecular entities and produces extensive gene lists with limited interpretability, differential network analysis has emerged as a crucial complementary analysis for comparative studies. Here, we present multiDEGGs (multiomics differentially expressed gene–gene pairs), a CRAN R package that enables differential network analysis in single or multiomic scenarios. **Methods:** multiDEGGs uses a multiomic graph framework where, for each data type, differential networks are generated and the statistical significance of each link is evaluated. These networks are then integrated into a comprehensive visualization that allows interactive exploration of cross-omic patterns and interactions. The package facilitates seamless integration into cross-validation machine learning pipelines for feature selection and identification of biologically relevant interactions for feature engineering. **Results:** We validated multiDEGGs using two cohorts of patients with rheumatoid arthritis. For each treatment group, multiomic differential interactions were identified, and eight machine learning models were trained to predict treatment resistance using synovial RNA sequencing data. We systematically compared multiDEGGs against seven feature selection methods. On average, area under the receiver operating characteristic curve values obtained with multiDEGGs showed an improvement of 0.10 compared to conventional filters. **Availability and Implementation:** multiDEGGs is freely available on CRAN, and the source code is available on GitHub at https://github.com/elisabettasciacca/multiDEGGs under GPL-3.0 license. Source code used to generate figures and analyses conducted in this paper is available at https://github.com/EMR-bioinformatics/multiDEGGs_supplementary.

## Highlights

- Traditional gene expression analysis leaves researchers with hundreds of “significant” genes but no clear biological story. The multiDEGGs CRAN package shifts the focus: Instead of asking which genes change, it asks which gene relationships change.
- It can be used with single or multiomic data: Differential networks are calculated separately for each data type, with results integrated into a comprehensive, interactive view.
- multiDEGGs can be combined with the nestedcv CRAN package (nested cross-validation) to serve as feature selection and augmentation tool.
- In comparative evaluations, machine learning models trained with multiDEGGs-selected features showed AUC improvements of 0.10 compared to other feature selection methods.

## Graphical Abstract

**Figure:**
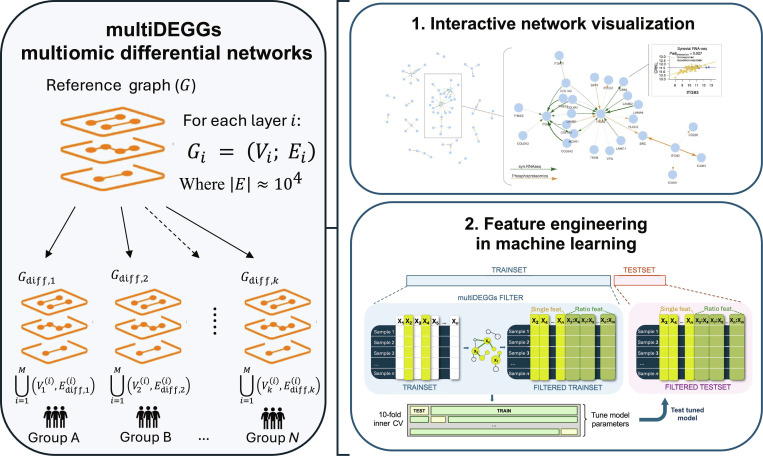

## Introduction

Modern clinical trials increasingly leverage high-throughput omics data to characterize distinct patient phenotypes and elucidate differential biological mechanisms across various conditions. In this context, differential gene expression analysis represents the most used analytical approach, despite its limitations in efficacy and interpretability [1–4]. Differential network analysis has emerged as a crucial complement to single-gene differential methods by identifying alterations in biomolecular relationships, thereby providing deeper insights into the molecular mechanisms underlying specific phenotypes or disease states.

The past decade has witnessed important advancements in the development of R packages dedicated to network analysis of high-throughput omics data. These tools can be broadly categorized on the basis of their statistical and mathematical strategies. One group of approaches primarily focuses on coexpression patterns derived directly from the omics data and identifying differentially coexpressed modules. Methods in this category typically construct networks by computing correlation measures, such as Pearson correlation, and subsequently compare them between conditions. No statistical test is applied to individual network edges; therefore, changes in the relationship between two molecules cannot be directly assessed. For example, the DiffCorr [5] package calculates correlation matrices and uses Fisher’s *z* test to identify differential correlations, while DiffCoEx [6] leverages the weighted correlation network analysis (WGCNA) framework to detect differentially coexpressed gene modules. WGCNA is also used—among other options—in the DCGL package [7], and the INDEED package [8] allows users to choose between partial, Pearson, and Spearman correlation.

In contrast to purely data-driven methods, another class of tools integrates prior knowledge of biological interactions to guide the network analysis. This strategy addresses the computational challenges of analyzing all possible feature combinations while enhancing interpretability.

For instance, DIABLO [9] integrates multiomics datasets to identify discriminatory molecular signatures while optionally structuring the analysis around pathway databases such as Kyoto Encyclopedia of Genes and Genomes (KEGG) or Reactome. Similarly, NetOmics [10] constructs temporal multilayer networks by combining data-driven inference algorithms (e.g., ARACNe [11]) with prior knowledge from interaction databases such as BioGRID and KEGG. However, these methods do not directly test the statistical significance of edge-specific changes under different conditions; rather, they identify differential signatures of individual molecules and subsequently represent them as networks without statistically evaluating the differential nature of the connections themselves.

DEGraph [12] represents a methodological shift toward explicit differential network analysis, although at the subgraph level. It performs multivariate tests (Hotelling’s test) to detect coordinated expression shifts within graph-defined subnetworks, using prior biological network topology to guide the identification of differential subgraphs between conditions. Nevertheless, DEGraph does not provide statistical assessment of individual edge changes, lacks interactive visualizations for network exploration, and offers no native support for machine learning workflows.

In our previous work, we introduced DEGGs (differentially expressed gene–gene pairs) [13], a method designed to detect differential interactions in single-omic scenarios, primarily transcriptomics, using classic or robust linear regression with interaction terms. DEGGs provided an interactive R Shiny interface enabling users to visually explore condition-specific differential networks and inspect the underlying regression models for individual edges.

Here, we present multiDEGGs (multiomics DEGGs), a substantial evolution of the original DEGGs framework that inherits its interactive exploration capabilities (Movie S1) while introducing key innovations: (a) the capability to handle and integrate heterogeneous multiomics data types within a unified multiomic network view that enables the identification of both intra-omic interactions (e.g., gene–gene and protein–protein) and cross-omic regulatory patterns (e.g., gene–protein mappings), revealing vertical signal propagation across molecular layers and tissue-specific multiomics associations; (b) significant algorithmic optimizations for computational speed, efficiency, and dependencies; and (c) native integration with nested cross-validation workflows for feature engineering in machine learning applications.

To support this latter functionality, we extended the nestedCV R package [14] with new capabilities for handling feature modifications across cross-validation folds.

We applied multiDEGGs to two rheumatoid arthritis (RA) case studies to identify multiomic differential networks that revealed key biological mechanisms distinguishing therapy responders from nonresponders, offering valuable insights for clinical investigation.

Furthermore, we demonstrated how the resulting differential interactions can be effectively leveraged for feature selection (and feature engineering) to predict treatment response. The features selected by multiDEGGs led to better model performances when compared with other well-known filters (e.g., univariate filters, glmnet regularization, etc.).

## Methodology

### Differential multiomic analyses through multilayer networks

**Fig. 1.**
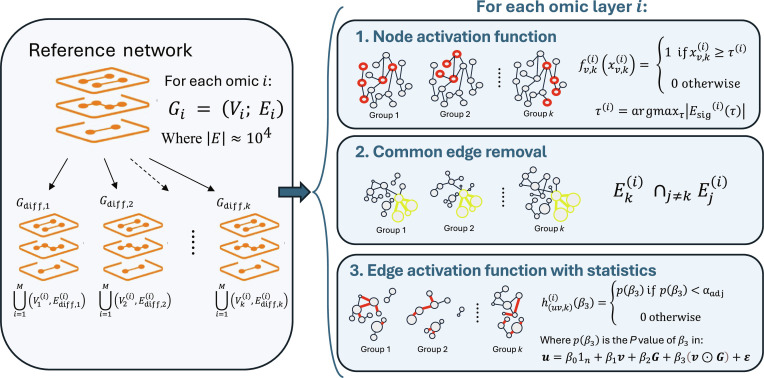
Workflow diagram showing the multiDEGGs internal implementation for multiomic differential network analysis.

The multiDEGGs package extracts differential multiomic graphs following the workflow depicted in Fig. 1.

In particular, let

i=1,…,M (1)

be a set of omic datasets available as input data and

k=2,…,K (2)

a set of experimental groups or conditions. Each dataset will define a layer in the multilayer structure and must be provided as a preprocessed expression matrix where appropriate quality checks and transformations have been applied according to the data type and experimental design. For example, for RNA sequencing (RNA-seq) data log_2_ or variance stabilizing transformation is recommended to stabilize the variance of data.

Let

$$G=\left(V;\;E\right)$$ (3)

be a reference biological network with vertex set V representing molecular entities and edge set E representing known molecular interactions from literature. The multiDEGGs package includes a human-specific reference network as default [15], containing approximately $\left|E\right|\approx 10^{4}$ edges. This network is derived from KEGG pathway interactions and enriched with microRNA and transcriptional regulatory interactions following the methodology described in [15]. While this default network supports human data analysis, users working with other species or requiring custom interaction sets can provide their own reference network through the get_diffNetworks() function, which accepts user-defined networks in tabular format, as detailed in the function documentation.

From the reference network G, multiDEGGs extracts group-specific differential multiomic graphs for each experimental group or condition :k

$$G_{\text{diff},k}=G_{\text{diff},k}^{\left(1\right)},G_{\text{diff},k}^{\left(2\right)},\ldots ,G_{\text{diff},k}^{\left(M\right)}$$ (4)

where each layer $G_{k}^{\left(i\right)}=\left(V_{k}^{\left(i\right)},\;E_{k}^{\left(i\right)}\right)$ represents the differential set of vertices and edges for a specific omic data type (e.g., RNA-seq, proteomics, O-link, etc.) filtered for group k.

The key distinction between groups arises from the group-specific activation patterns of molecular entities.

During step 1, for each group k and layer i, multiDEGGs defines a node activation function $f_{v,k}^{\left(i\right)}:R\to 0,1$ as a step function:

fv,kixv,ki=1ifxv,ki≥τi0otherwise (5)

Here, $x_{v,k}^{\left(i\right)}$ represents the normalized median expression or abundance of the biological entity v in group k within the omic layer i, and $\tau^{\left(i\right)}$ is the activation threshold selected by solving:

$$\tau^{\left(i\right)}=\text{argma}x_{\tau }\left|{E_{\mathrm{sig}}}^{\left(i\right)}\left(\tau \right)\right|$$ (6)

where ${E_{\mathrm{sig}}}^{\left(i\right)}\left(\tau \right)$ is the set of significant edges at threshold τ, as per percolation procedure detailed in the following paragraph. The group-specific active vertex set for layer i is then defined as

$$V_{k}^{\left(i\right)}=v\in V:f_{v,k}^{\left(i\right)}\left(x_{v,k}^{\left(i\right)}\right)=1$$ (7)

Since $x_{v,k}^{\left(i\right)}$ varies between groups, during the first step, different nodes will be activated for each group k, creating group-specific network topologies.

To retain only the group-specific edges, in step 2, multiDEGGs identifies and removes edges that are common to all k groups within each omic layer. For layer i, the group-specific edge set is then defined as

$$E_{k,\text{specific}}^{\left(i\right)}=E_{k}^{\left(i\right)}\;\cap_{j\neq k}E_{j}^{\left(i\right)}$$ (8)

where $E_{k}^{\left(i\right)}$ represents the edges connecting active nodes in group k and layer i.

In step 3, an edge activation function is applied to each remaining group-specific edge in layer i.

multiDEGGs uses linear regression with interaction term to assess whether the differential connectivity between groups is statistically significant. In general, adding an interaction term to a linear model entails modeling not only how one variable depends on another but also how this relationship can be influenced by a third variable, which, in this case, is the categorical variable indicating membership to a phenotypical group or experimental condition. Statistical significance (*P* value) associated with this interaction term indicates differential dependency between two molecular entities across groups (e.g., coactivation in one group but inhibition or absence of correlation in another).

For each potential edge $\left(u,\;v\right)$ connecting active nodes within the same layer i and group k, let $u,v\in R^{n}$ be the corresponding normalized expression or abundance vectors of the two biological entities across all samples and $k\in 2,\ldots ,K^{n}$ be the categorical group vector indicating group membership for each sample.

For the 2-group case (K=2), multiDEGGs formulates the regression model as follows:

$$u=\beta_{0}1_{n}+\beta_{1}v+\beta_{2}k+\beta_{3}\left(v⊙k\right)+\varepsilon$$ (9)

where $1_{n}$ is the vector of ones of dimension n, ⊙ denotes the element-wise (Hadamard) product, $\beta_{3}$ captures the differential interaction effect between the two biological entities in group k relative to other groups, and $\varepsilon \in R^{n}$ represents the error term vector.

The model can be fitted using either ordinary least-squares regression (lm) or robust linear regression (rlm) to handle potential outliers.

For the multigroup case (K≥3), an ANCOVA (analysis of covariance) framework is used with the same model structure, where the *F* test for the interaction term $\left(v⊙k\right)$ evaluates whether the association between the two features differs across the *K* groups. This allows detection of differential connectivity patterns when comparing three or more experimental conditions.

In both cases, the null hypothesis $H_{0}:\beta_{3}=0$ is tested against the alternative $H_{1}:\beta_{3}\neq 0$.

The differential edge activation function $h_{\left(\mathrm{uv},\;k\right)}^{\left(i\right)}:\to R$ is then defined as

huvkiβ3=pβ3ifpβ3<αadj0otherwise (10)

where $p\left(\beta_{3}\right)$ is the adjusted *P* value for the interaction term $\beta_{3}$ and $\alpha_{\mathrm{adj}}$ is the multiple testing corrected significance threshold defined by the user.

It is important to note that the multiple testing correction is applied independently for each set of tested edges $\left(u,\;v\right)$ within the same layer i and group k. By default, the Bonferroni correction is applied; however, users can specify alternative methods such as Benjamini–Hochberg (false discovery rate), Storey’s, or others, depending on the desired control of type I error rate.

The differential edge set for layer i is then defined as follows:

$$E_{\left(k,,\text{diff}\right)}^{\left(i\right)}=\left(u,,v\right)\in E_{\left(k,,\text{specific}\right)}^{\left(i\right)}:h_{\left(\mathrm{uv},,k\right)}^{\left(i\right)}\left(\beta^{3}\right)>0$$ (11)

Therefore, the complete group-specific differential multiomic graph is represented as follows:

$$G_{\text{diff},k}=\overset{M}{\underset{i=1}{⋃}}G_{\text{diff},k}^{\left(i\right)}$$ (12)

where $G_{\text{diff},k}^{\left(i\right)}=\left(V_{k}^{\left(i\right)},\;E_{\text{diff},k}^{\left(i\right)}\right)$ is the differential graph for group k and layer i.

It is worth noting that, in this final representation, each molecular entity is active in only a subset of omic layers.

For visualization purposes, the final multiomic differential networks are integrated into a comprehensive display, where each omic layer i is assigned a distinct color, enabling users to easily gain an overview of all omic data and explore cross-omic patterns.

The interactive visualization is implemented through the View_diffNetworks()function using R Shiny and includes several features: a slider for filtering network links by *P* value (or adjusted *P* value), a search box to locate specific genes or molecular entities, clickable links that display the associated differential regression plot when selected, and clickable nodes that generate box plots showing expression differences between experimental groups (Movie S1).

It is important to highlight that, as described above, multiDEGGs implements a modular architecture where each omic layer is processed independently before integration at the visualization stage. This design choice provides several methodological advantages for handling data heterogeneity, as the 3-step procedure (node activation, common edge removal, and edge activation) is applied independently to each omic dataset without requiring cross-omic sample matching. Consequently, the framework does not impose requirements for complete omic profiles for all subjects and uniform normalization protocols across different omics. No imputation or sample exclusion is performed by multiDEGGs. Similarly, batch effect correction and other preprocessing steps (normalization and transformation) are not performed internally but must be applied to each dataset prior to analysis. The multiomic structure is established at the final stage by stacking the independently derived networks and aligning nodes across layers based on known biological mappings (e.g., gene–protein correspondences). This visualization enables the identification of interomic regulatory patterns and vertical signal propagation.

### Adaptive thresholding through percolation analysis

When analyzing experimental data, threshold-based filtering is necessary to remove low, noisy signals but is inherently sensitive to arbitrary cutoff choices. To mitigate this arbitrariness, multiDEGGs implements an adaptive heuristic to fine-tune the τ threshold parameter and maximize the signal-to-noise ratio of the resulting network.

This approach is grounded in percolation analysis, a framework from network theory [16], in which a network parameter is systematically varied to characterize emergent structural properties and phase transitions [16]. In classical percolation, edges or nodes are progressively added (or removed) to observe how global connectivity evolves. Here, we adapt this concept by varying the mean expression threshold τ across a range of quantile values (from the 35th percentile onward) and monitoring the interplay between network size and statistical signal strength.

Biological networks, similar to other complex networks, undergo a phase transition between a fragmented state (at high thresholds, where few nodes are active) and a dense, overconnected “hairball” state (at low thresholds, where spurious connections dominate). Our heuristic identifies the optimal balance by weighing network connectivity against the statistical penalty imposed by multiple hypothesis testing. As the threshold decreases, the inclusion of more nodes increases the stringency of multiple testing correction. The algorithm identifies the peak where the inclusion of additional nodes yields a net increase in significant edges despite the stricter penalty, ensuring that the gain in biological signal outweighs the statistical cost of noise.

Empirical analyses show that below the 30th percentile, networks systematically enter an unstable noise region characterized by the hairball effect. To prevent users from entering this unstable regime, multiDEGGs implements a conservative default minimum scanning threshold of 0.35 (35th percentile).

### Benchmarking with simulated data

To validate the performance of multiDEGGs in detecting differential coexpression patterns, we conducted a comprehensive benchmarking analysis using simulated molecular data with known ground truth. Specifically, correlations in synthetic datasets were generated with varying levels of noise (SD = 0.5, 0.8, and 1.2), outlier contamination (10%, 20%, and 30%), and interaction strength (*β*_3_ = 0.5, 0.75, and 1.0) to assess sensitivity and specificity across realistic data quality scenarios. This benchmarking analysis demonstrated that multiDEGGs maintains excellent specificity (>99%) across all conditions, effectively controlling false-positive rates. Sensitivity varied with signal strength and data quality. Under optimal conditions (low noise and few outliers), sensitivity reached 96.5% to 100% even for moderate interaction effects. Under challenging conditions (high noise and 30% outliers), sensitivity remains >88% for strong signals (*β*_3_ ≥ 0.75), but decreases to ~59% for weak signals (*β*_3_ = 0.5).

Despite the apparent loss in performance, this conservative approach prevents multiDEGGs from potential overinterpretation of weak differences. Full details of the simulation framework, parameter settings, and performance metrics are provided in Supplementary Methods, Figs. S1 and S2, and File S1.

### multiDEGGs for feature engineering in machine learning applications

In clinical settings, high-throughput molecular data are increasingly collected not only to elucidate disease biology but also to predict patient-specific outcomes, such as treatment response or disease progression. Beyond their role in biomarker discovery and hypothesis generation, the differential interactions identified by multiDEGGs can also be leveraged for this predictive purpose in machine learning applications. Because of the high-dimensional nature of these data, a key challenge is that the number of predictors (*P*) substantially exceeds the sample size (*s*). This dimensional imbalance requires selective feature inclusion, both for mathematical and clinical reasons. Models trained in *P* ≫ *s* conditions are prone to overfitting, resulting in high variance, poor generalizability, and spurious correlations. Second, the use of all variables available in high-throughput data would not be feasible in clinical practice beyond research studies. For example, the implementation of targeted panels with selected, predictive biomarkers represents a more feasible approach than collecting whole-transcriptome RNA-seq in clinical routine.

Traditional feature selection approaches focus on identifying individual predictors that demonstrate the strongest association with the outcome of interest (e.g., univariate filters such as *t* test or Wilcoxon test). However, since biological systems operate through complex networks of interacting components, high predictive power may also reside in feature combinations and interactions. In general, feature engineering involves a set of techniques that enables the creation of new features by combining or transforming the existing ones [17,18]. For example, interaction terms are usually captured by multiplying or dividing two or more original features, while polynomial features extend this concept to include powers of individual variables and their combinations. Such a mathematical framework allows models to capture complex, nonadditive relationships where the effect of one variable depends on the value of another. The use of combined predictors and polynomial transformations thus offers important potential to enhance machine learning models because they enable the integration of nonlinear biological mechanisms that individual features fail to represent.

The higher-order informative content carried by differential interactions, combined with the multiDEGGs’ ability to extract only differential links validated in literature, positions it as particularly suitable both for single feature selection and for guiding the creation of modified predictors. Specifically, this addresses a key limitation of conventional black-box algorithms, which may select single predictors lacking biological significance and thus compromise the credibility and explainability of the resulting model in clinical settings.

The first version of this package (DEGGs) has already demonstrated potential in identifying gene–gene interactions predictive of treatment response in RA [19]. However, the validation of its efficacy, as both a feature selection method and a predictor modification strategy, requires evaluation in larger cohorts with rigorous cross-validation protocols.

### Implementation of functions for feature engineering in cross-validation frameworks

**Fig. 2.**
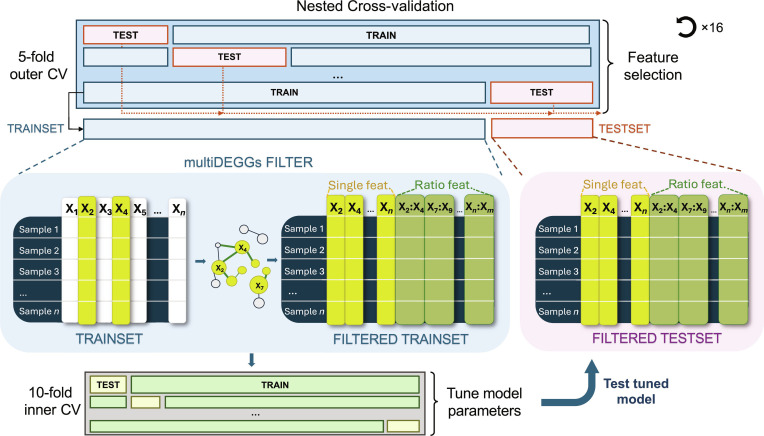
Nested cross-validation machine learning framework using multiDEGGs-based feature engineering. The outer 5-fold cross-validation (CV) splits the data into training and test sets. For each outer fold, the multiDEGGs filter is applied to the training set to identify differential network edges. The TRAINSET matrix (left) contains all original features (X_1_, X_2_, ..., X*_n_*). Differential network analysis identifies pairwise interactions (e.g., X_2_–X_4_), which are used to generate the FILTERED TRAINSET matrix containing: (a) individual features involved in differential edges (shown in yellow, e.g., X_2_ and X_4_) and (b) ratio-based features computed from each edge (shown in green, e.g., X_2_:X_4_). The same transformation is applied to the independent test set, producing the FILTERED TESTSET with the same feature structure. An inner 10-fold cross-validation on the filtered training set is used to tune model hyperparameters, and the final tuned model is evaluated on the filtered test set. This process is repeated across all outer folds to obtain robust performance estimates. The entire nested cross-validation procedure is repeated 16 times in our case study.

Previous research has shown that applying filtering across the entire dataset introduces bias when assessing model accuracy [20]. Feature selection and predictor modification must be conducted exclusively on training data to prevent information leakage from the test set. The traditional holdout approach partitions the dataset into two-thirds training and one-third test, with filtering performed only on the training portion. However, even more robust estimates can be obtained if the holdout set is generated multiple times through random splits. In such a nested cross-validation setting, performance metrics can be aggregated to provide more reliable measurements. In our framework, the outer cross-validation loop repeatedly partitions the data into training and test sets. For each outer fold, the multiDEGGs filter is applied exclusively to the training data to identify differential network edges, which are then used to extract both individual and ratio-based features. This filtered feature set is used for model training via an inner cross-validation loop for hyperparameter tuning, and the same feature transformation (single and ratio-based features learned from the training data) is applied to the independent test set for evaluation. This process ensures that feature engineering is performed independently within each outer fold, preventing information leakage and providing unbiased performance estimates (Fig. 2).

The nestedCV package available on CRAN [14] allows automatic implementation of nested cross-validation for feature selection, but its original version did not include functionality for nested feature engineering. To address this methodological requirement, we have expanded its code to enable the nested modification of predictors within each outer fold, ensuring that the attributes learned from the training part are applied to the test data without prior knowledge of the test data itself. The selected and combined features, as well as corresponding model, can then be evaluated on the holdout test data without introducing bias.

**Fig. 3.**
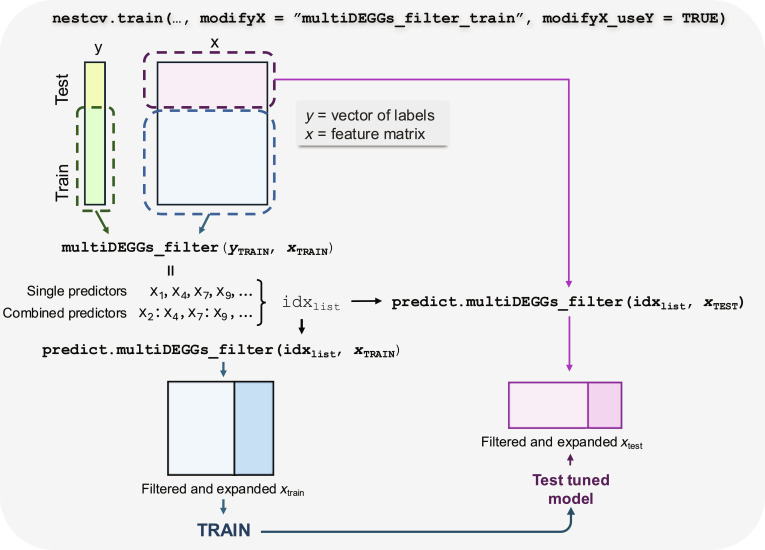
Code architecture for integrating multiDEGGs-based feature processing with the nestedcv cross-validation framework. The nestedcv.train()function receives the outcome vector (*y*) and feature matrix (*x*) from the outer cross-validation loop. When modifyX = "multiDEGGs_filter_train", the multiDEGGs_filter() function is called on the training data (*y*_TRAIN_, *x*_TRAIN_) to identify differential network edges. This function returns a list object (idx_list) containing the indices of (a) single predictors involved in differential edges (X_1_, X_4_, X_7_, X_9_, ..., shown in yellow in Fig. 2) and (b) combined predictors representing ratio-based features (X_2_:X_4_, X_7_:X_9_, ..., shown in green in Fig. 2). The predict.multiDEGGs_filter()method then applies this feature transformation to both training and test sets, creating matrices with individual features and their corresponding ratios (e.g., X_2_:X_4_). The filtered and expanded matrices are used for model tuning (inner cross-validation) and final evaluation.

Specifically, the nestedCV code has been extended to accept any user-defined function that filters or transforms the feature matrix by passing the function name to the modifyX parameter of the nestcv.train()function. A Boolean flag (modifyX_useY) determines how the transformation is applied. When set to TRUE, the transformation is fitted using both the training response labels (i.e., the dependent variable) and the training feature matrix. The fitted model is then stored and used by another user-defined predict()function to modify the feature space in both training and testing data (Fig. 3).

This approach is suitable for supervised transformations that need to learn from the training data, such as principal components analysis or the differential network analysis discussed in this paper. For unsupervised transformations such as scaling, normalization, or polynomial feature generation that do not require knowledge of the response variable, modifyX_useY must be set to FALSE.

For this implementation, we then defined—and included in the multiDEGGs package—the filtering and predict functions as follows.

The multiDEGGs_filter()function identifies the differential molecular interactions, extracts both individual and paired corresponding variables, and stores them into an S3 model object containing the indices of each selected feature. Under this scheme, the gene expression threshold used to filter low-expressed genes (internally determined by multiDEGGs via percolation analysis) is estimated independently within each training fold of the outer cross-validation loop. This ensures that threshold selection does not use any information from the corresponding test fold, thereby preventing data leakage and maintaining the validity of performance estimates.

The second function [predict.multiDEGGs_filter()] is the predict method that applies the feature selection learned from the training set to new data. Specifically, it takes the list of paired variables identified during training and creates ratio-based features (feat. A:feat. B), while also retaining the individual ones involved in the differential interaction (i.e., feat. A and feat. B alone). This ensures that both training and test data of each outer fold undergo the same feature transformation based on the patterns learned from the training set (Fig. 3).

Finally, it is also worth noting that while traditional filtering functions require the number of selected features to be set by the user, multiDEGGs automatically establishes the best number of predictors due to the internal percolation process. Therefore, although a maximum threshold can still be set to prevent excessive feature inclusion, the exact number of selected features is automatically fine-tuned, eliminating arbitrary decision-making.

### Package dependency analysis and optimization

The complexity of package dependencies has emerged as a critical consideration in R package development, particularly within the bioinformatics ecosystem where numerous interdependent packages can significantly impact installation time, maintenance burden, and computational resource requirements [21]. Dependency heaviness—defined as the number of additional packages that a parent uniquely brings to a child package—provides a quantitative framework for assessing and optimizing package dependency structures. To evaluate and minimize the dependency burden of our package, we used the pkgndep R package (v1.99.3) [21], which systematically analyzes packages listed in the Depends, Imports, LinkingTo, and Suggests fields of the DESCRIPTION file of a package. The initial dependency analysis performed on the previous implementation of the code (DEGGs package) revealed a complete dependency tree with 86 packages directly required for installation (Fig. S3). For the development of multiDEGGs, we identified potential optimization targets and reduced the dependency heaviness by replacing heavy parent packages with lighter alternatives and removing nonessential dependencies, resulting in a substantially streamlined package architecture with 54 packages directly required (Fig. S4). This optimization process not only reduces installation time and potential for dependency conflicts but also enhances the package’s long-term maintainability and accessibility for end users.

## Results

### Multiomic differential network analysis in RA

We applied multiDEGGs to two RA multiomic datasets. RA is a chronic autoimmune disease characterized by synovial inflammation and joint destruction. Despite the availability of diverse biologic disease modifying antirheumatic drugs targeting distinct immune pathways, 30% to 40% of patients with RA exhibit inadequate response [22], and approximately 10% develop multirefractory disease, failing more than two biologic classes in longitudinal studies [23]. To better understand the biological signatures underlying treatment response, we applied multiDEGGs to two independent and nonoverlapping cohorts of patients with RA who underwent, for the first time, tocilizumab and rituximab therapy, respectively [24–29].

In both studies, ultrasound-guided synovial biopsies were collected before treatment initiation, and therapeutic response was assessed following treatment completion. The resulting multilayer differential networks highlighted important mechanisms distinguishing patients who respond to therapy from those who do not, providing crucial insights for clinical investigation.

### Multiomic differential network analysis in patients with RA not responding to tocilizumab therapy

The RA cohort of patients treated with tocilizumab consisted of 65 individuals with available synovial RNA-seq data (*n* = 65), O-link proteomic data (*n* = 64), and lower numbers of mass spectrometry proteomic (*n* = 17) and phosphoproteomic profiles (*n* = 17) [24,30] (see Supplementary Methods for details and preprocessing of data).

The internal percolation analysis identified optimal filtering thresholds of 70th percentile for RNA-seq, 45th percentile for O-link, and 35th percentile for both mass spectrometry proteomic and phosphoproteomic layers. To show that the selected thresholds are robust relative to random thresholds, we performed a permutation test on the RNA-seq layer (*n* = 50 iterations). The peak of observed network connectivity (70th percentile) clearly distinguished from the random noise distribution that dominated only at substantially lower thresholds (Fig. S5A).

**Fig. 4.**
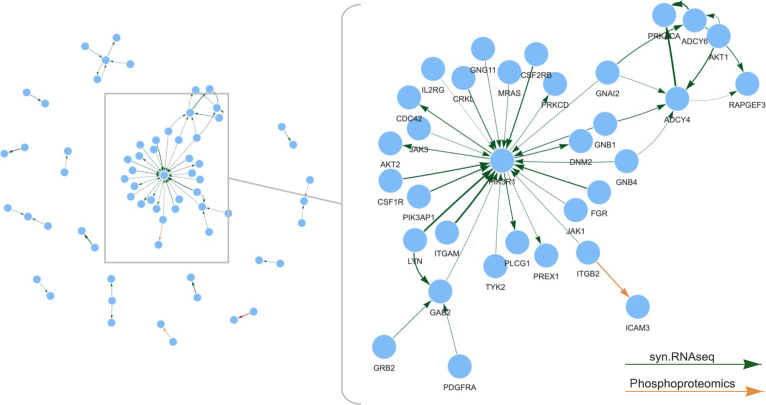
Network overview and magnified view of a cluster of multiomic differential interactions between tocilizumab responders and nonresponders in patients with RA. The network displays differential interactions with edge colors representing different omics data types (green edges for synovial RNA-seq [syn.RNAseq] and orange edges for phosphoproteomics data). All interactions shown have adjusted *P* < 0.05. Edge thickness is proportional to statistical significance, with thicker edges representing lower adjusted *P* values. Arrow directions reflect literature-reported regulatory relationships and are not derived from data.

The resulting multiomic differential network comprised 67 nodes and 59 edges across the 4 molecular omics. The main cluster centers on *PIK3R1*, which shows extensive synovial RNA-seq coexpression (green arrows) with key cytokine-driven inflammatory pathway signaling molecules including *JAK1*, *JAK3*, *TYK2*, *IL2RG*, *CSF2RB*, and *AKT2* (Fig. 4). This indicates possible coordinated activation of the phosphatidylinositol 3-kinase–AKT and Janus kinase (JAK)/signal transducers and activators of transcription pathways, both of which are downstream of cytokine receptors, including the interleukin-6 receptor (IL-6R) [31]. The coexpression of *ADCY4* and *PRKACA* (adjusted *P* = 0.0074) in responders suggests coordinated activation of the cyclic adenosine monophosphate (cAMP)–protein kinase A pathway. *ADCY4* encodes adenylate cyclase 4, which generates cAMP as a second messenger, while *PRKACA* encodes the catalytic subunit of protein kinase A, the primary effector of cAMP signaling. Together, their coexpression implies enhanced cAMP-driven phosphorylation of downstream targets, which is known to increase secretion of cytokines including IL-6 [32]. Phosphoproteomic links (orange arrows) involving integrin subunit β2 (ITGB2) and intercellular adhesion molecule 3 (ICAM3) suggest potential integrin-mediated adhesion and immune cell retention pathways. However, given the limited sample size of the phosphoproteomic layer (*n* = 17), these findings should be interpreted cautiously. A bootstrap-based stability analysis (see Supplementary Methods and Fig. S6A) suggested that edges from this layer show lower reproducibility, reflecting reduced statistical power.

From a clinical perspective, the presence of this integrated network prior to therapy suggests that the pathology in responders is highly dependent on cytokine, including IL-6, mediated phosphatidylinositol 3-kinase–AKT, and JAK/signal transducers and activators of transcription signaling for synovial inflammation. Tocilizumab, by blocking IL-6R signaling, likely disrupts this central network, leading to a more profound therapeutic effect in these patients. Conversely, the absence of this transcriptional and phosphoproteomic configuration in nonresponders suggests that their synovial inflammation is driven by alternative, IL-6-independent pathways.

### Dysregulated multiomic interactions in patients with RA treated with rituximab

The RA cohort of patients receiving rituximab treatment comprised 72 patients with accessible synovial RNA-seq along with fewer mass-spectrometry-based proteomic and phosphoproteomic profiles (*n* = 21) [26,30] (see Supplementary Methods for details on preprocessing of data).

The internal percolation analysis identified optimal filtering thresholds of 70th percentile for RNA-seq and 50th and 40th percentiles for proteomic and phosphoproteomic layers, respectively. Permutation testing on the RNA-seq layer (*n* = 50 iterations) also confirmed for this cohort that the peak of network connectivity (70th percentile) is clearly separated from the random noise distribution (Fig. S5B).

**Fig. 5.**
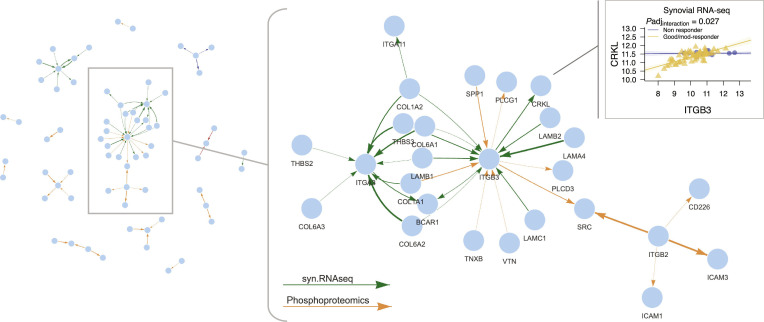
Network overview with magnified view of a cluster of multiomic differential interactions between rituximab responders and nonresponders in patients with RA. The network displays differential interactions with edge colors representing different omics data types (green edges for synovial RNA-seq and orange edges for phosphoproteomics data). All interactions shown have adjusted *P* (*P*adj) < 0.05. Edge thickness is proportional to statistical significance, with thicker edges representing lower adjusted *P* values. Arrow directions reflect literature-reported regulatory relationships and are not derived from data.

The resulting multiomic differential network (64 nodes and 60 edges) highlights *ITGB3* and *ITGA3* as central genes (Fig. 5). They encode integrin subunits β3 and α3 that mediate cell–extracellular matrix (ECM) adhesion and signaling, processes central to synovial fibroblast activation and immune cell trafficking in RA [33].

Their presence suggests integrin-mediated ECM interactions, which represent a key distinguishing factor for rituximab responders in RA. *ITGB3* expression is strongly associated with multiple ECM component genes (*LAMA4*, *LAMB1*, *LAMB2*, *COL1A1*, *COL6A2*, *THBS3*, and *SPP1*) in responders, consistent with its role in modulating fibroblast adhesion, migration, and tissue remodeling in the synovium. Notably, the association of ITGB3 with proto-oncogene tyrosine-protein kinase Src (SRC) and phospholipase C-γ1 (PLCG1) through phosphoproteomics data suggests downstream activation of focal adhesion and mitogen-activated protein kinase signaling pathways, which regulate proinflammatory and survival signals. Through SRC, this phosphoproteomic network extends to ITGB2, a leukocyte-specific integrin central to immune cell adhesion and migration [33]. The ITGB3–SRC–ITGB2 axis may facilitate immune cell, particularly B cell, recruitment into the inflamed synovium, aligning with the higher B cell burden observed in rituximab responders [24]. However, as with the tocilizumab cohort, the limited sample size (*n* = 21) results in lower stability for phosphoproteomic edges (Fig. S6B), and these interactions should be considered exploratory. The top right plot demonstrates a significant positive correlation between *ITGB3* and *CRKL* (which encodes an adaptor protein involved in integrin and growth factor signaling) expression in good/moderate responders but absent in nonresponders. The strong coupling of *ITGB3–CRKL* expression in responders indicates that integrin-mediated signaling dynamics, potentially linked to cellular adhesion and immune cell trafficking, are more transcriptionally coordinated in patients who benefit from rituximab.

The integrin subunit α3 (ITGA3) network also shows strong coexpression with ECM-related genes, including *COL1A1*, *COL1A2*, *COL6A1*, *COL6A2*, *COL6A3*, *LAMB1*, *THBS2*, and *THBS3*, indicating active matrix remodeling and cell–matrix adhesion in rituximab responders. This pattern suggests that fibroblast-like synoviocytes are engaged in strong integrin–ECM interactions, promoting a structural microenvironment conducive to immune cell infiltration and retention.

Collectively, these networks may indicate that responders possess a synovial microenvironment preconditioned for immune cell (including B cell) migration, retention, and survival via integrin–ECM interactions. As rituximab targets CD20^+^ B cells, its efficacy is enhanced in B-cell-rich synovium, which may explain why responders display an *ITGB3/ITGA3*-centered network of transcriptional and phosphoproteomic associations that is absent in nonresponders.

### Feature selection for treatment response prediction

As detailed in Methodology section, multiDEGGs can be also used as feature engineering method in machine learning pipelines. To evaluate the impact of multiDEGGs feature engineering on machine learning performance, we trained eight machine learning models to predict treatment resistance using synovial RNA-seq data from the two cohorts. The models used were as follows: gradient boosting machine (gbm), generalized linear model with elastic net regularization (glmnet), penalized discriminant analysis (pda), partial least-squares (pls), random forest (rf), support vector machine with polynomial kernel (svmPoly), extreme gradient boosting with linear booster (xgbLinear), and extreme gradient boosting with tree booster (xgbTree).

For each model, we systematically compared multiDEGGs against 7 feature selection methods: Wilcoxon rank-sum test, ReliefF, partial least-squares (PLS), random forest, Boruta, *t* test, and generalized linear model with elastic net regularization (glmnet. This selection was designed to include widely adopted filters in transcriptomic analysis along with methods with advanced capabilities comparable to multiDEGGs. Three filtering methods share multiDEGGs’ ability to capture feature interactions: Random forest captures nonlinear interactions through ensemble tree structures, Boruta uses a random forest-based wrapper to evaluate features in multivariate context, and ReliefF assesses features based on nearest-neighbor discriminative power. Two filters implement automatic feature selection as multiDEGGs does: glmnet via cross-validation-based regularization and Boruta through comparison with shadow features. To ensure fair comparison, we set the same maximum number of final features for all eight filtering approaches (40 for the tocilizumab cohort and 50 for the larger rituximab cohort).

Finally, to guarantee robust evaluation, each model/filter combination has been trained 16 times using nested cross-validation with 5 outer folds and 10 inner folds following the schema depicted in Fig. 2.

**Fig. 6.**
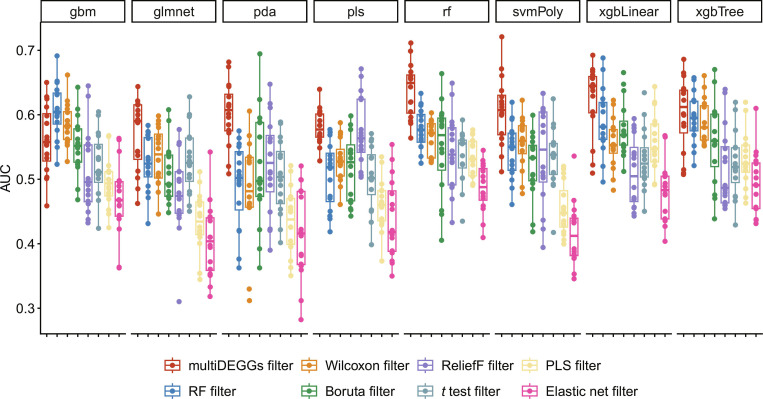
Box plots showing AUC values of each trained model in the prediction of the tocilizumab resistant state of patients with RA. Eight different models were tested: gradient boosting machine (gbm), generalized linear model with elastic net regularization (glmnet), penalized discriminant analysis (pda), partial least-squares (pls), random forest (rf), support vector machine with polynomial kernel (svmPoly), extreme gradient boosting with linear booster (xgbLinear), and extreme gradient boosting with tree booster (xgbTree). For each model, multiDEGGs was systematically compared against seven filtering methods: Wilcoxon rank-sum test, ReliefF, partial least-squares (PLS), random forest, Boruta, *t* test, and generalized linear model with elastic net regularization. The maximum number of selected features was set to 40 for all filters.

**Fig. 7.**
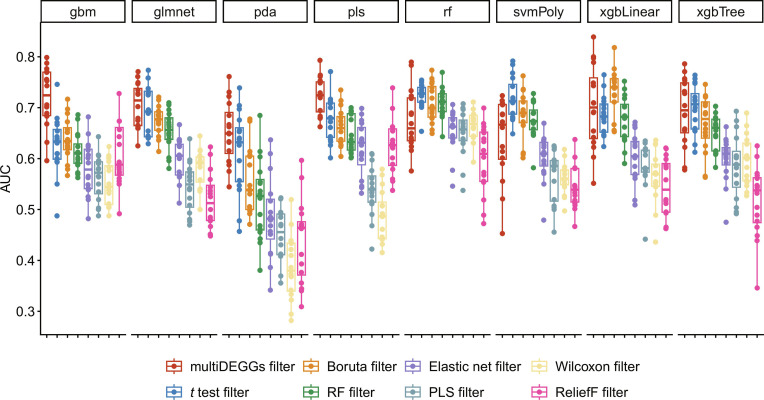
Box plots showing AUC values of each trained model in the prediction of the rituximab resistance for patients with RA. Eight different models were tested: gradient boosting machine (gbm), generalized linear model with elastic net regularization (glmnet), penalized discriminant analysis (pda), partial least-squares (pls), random forest (rf), support vector machine with polynomial kernel (svmPoly), extreme gradient boosting with linear booster (xgbLinear), and extreme gradient boosting with tree booster (xgbTree). For each model, multiDEGGs was systematically compared against seven filtering methods: Wilcoxon rank-sum test, ReliefF, partial least-squares (PLS), random forest, Boruta, *t* test, and generalized linear model with elastic net regularization. The maximum number of selected features was set to 50 for all filters.

Figures 6 and 7 report the area under the receiver operating characteristic curve (AUC) for both tocilizumab and rituximab cohorts. Each point represents an individual trained model from one of the 16 cross-validation repetitions for a specific model/filter combination (*n* = 16 points per combination), with no aggregation applied across models.

Figures S7 and S8 show equivalent plots reporting accuracy and balanced accuracy values for both datasets. On average, starting from 22,975 protein-coding genes available in synovial RNA-seq, multiDEGGs reduced the feature space to 29 predictors for the tocilizumab cohort and 30 features for the rituximab cohort. Model performances obtained with multiDEGGs consistently outperformed the models trained with the alternative feature selection methods, particularly when using random forest and support vector machines. On average, AUC values obtained with multiDEGGs incremented by 0.10. All AUC, accuracy, and balanced accuracy values, with corresponding 95% confidence intervals and SDs, are reported in File S2.

To ensure that this performance improvement is not inflated because of the presence of predictors as both single and combined variables, we repeated the same analysis switching to FALSE the keep_single_genes parameter of the predict.multiDEGGs()function. This ensures that only combined predictors—derived from differential pairs—are selected as features and the list of single variables stored by multiDEGGs_filter_train()function is ignored. As the feature space considerably decrease in this case, the filtering threshold was set to 15 for the tocilizumab cohort and to 25 for the larger rituximab group. Although its overall predictive power decreased as a consequence of the smaller feature space, multiDEGGs confirmed better feature selection in most models (Fig. S9 and File S3). In particular, in the tocilizumab, cohort models trained with multiDEGGs obtained higher AUC values in 5 of 8 models, while the random forest filter’s AUC values were comparable or slightly higher for the remaining models. On the other hand, the rituximab dataset showed higher performances in 3 of 8 models but performed poorly for the remaining models (gradient boosting machine, random forest, support vector machine with polynomial kernel, extreme gradient boosting with linear booster, and extreme gradient boosting with tree booster), indicating higher sensitivity to feature space reduction and insufficient filtering power with fewer features.

## Limitations

Some limitation must be acknowledged regarding both the methodology and data analysis presented in this study. These relate primarily to the dependency on reference network quality, the impact of sample size heterogeneity across omic layers, and the challenges of cross-cohort validation in RA molecular studies.

First, it must be clarified that the quality of differential networks identified by multiDEGGs depends fundamentally on the reference biological network used. The package provides a default human reference network (~10^4^ interactions) suitable for human studies, but users analyzing nonhuman organisms or requiring specialized contexts must provide appropriate reference networks from relevant databases. Since multiDEGGs can only detect interactions present in the reference network, incomplete or biased networks may lead to missed biological relationships or spurious associations. Users should carefully evaluate the source, coverage, and evidence levels of their chosen reference network and document these characteristics when reporting results from multiDEGGs.

In addition, the reliability of differential network inference also depends on sample size and data quality across omic layers. While multiDEGGs can integrate datasets with heterogeneous sample sizes, users should evaluate the robustness of omic-specific findings and, when possible, perform stability assessments. In our analysis, the proteomics and phosphoproteomics layers had limited sample numbers (*n* = 17 and *n* = 21), resulting in reduced statistical power and lower edge stability.

Finally, while our nested cross-validation approach provides robust performance estimates within each cohort, it must be acknowledged that external validation could not be assessed. The two RA cohorts analyzed represent distinct patient populations at different disease stages, treated with drugs having fundamentally different mechanisms of action (rituximab, a B-cell-depleting agent, versus tocilizumab, an IL-6 receptor inhibitor). Cross-cohort validation (e.g., training on tocilizumab-treated patients and testing on rituximab-treated patients) would be inappropriate, as the response to one mechanism cannot predict response to another. External validation would require comparable independent cohorts treated with the same drugs. Unfortunately, multiomic datasets in RA remain scarce, and the two cohorts used here represent some of the few available resources with sufficient sample size and quality for machine learning analysis. Future studies should prioritize collecting independent validation cohorts to more robustly assess the generalizability of our results.

## Conclusion

multiDEGGs performs multiomic differential network analysis by revealing differential interactions between molecular entities (genes, proteins, transcription factors, or other biomolecules). For each omic dataset provided, a differential network is constructed where links represent statistically significant differential interactions between biological entities. These networks are then integrated into a comprehensive visualization that allows interactive exploration of cross-omic patterns, such as differential interactions present at both transcript and protein levels. For each link, users can access differential statistical significance metrics (*P* values or adjusted *P* values) and differential regression plots.

multiDEGGs can also be used as feature selection/augmentation tool in machine learning pipelines. Models trained with features engineered by multiDEGGs constantly improved their performances compared to traditional filters.

## Data Availability

All RNA-seq data used in this article are available in ArrayExpress (https://www.ebi.ac.uk/biostudies/arrayexpress) and can be accessed with accession ID E-MTAB-13733 and E-MTAB-11611. Both multiDEGGs and nestedCV R packages are available on CRAN. Source code used to generate figures and analyses conducted in this paper is available at https://github.com/EMR-bioinformatics/multiDEGGs_supplementary.
