# Supplementary material for: multiDEGGs: Single or Multiomic Differential Network Analysis for Biomarker Discovery and Feature Engineering for Predictive Modeling
Source: Comput Struct Biotechnol J. 2026 Mar 18;35(1):0001. doi: 10.34133/csbj.0001 (PMC13082464; doi:10.34133/csbj.0001)
Supplement: Supplementary 1 — Supplementary Methods Figs. S1 to S9 Files S1 to S3 Movie S1 [file csbj.0001.f1.zip › Supplementary figures.pdf]

# SUPPLEMENTARY FIGURES

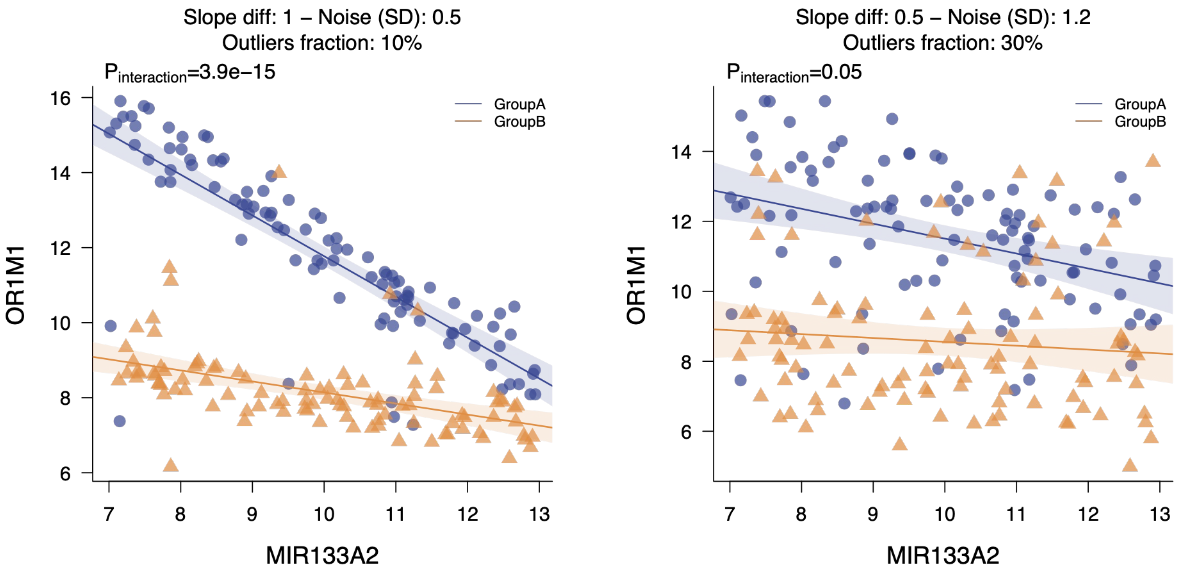


**Supplementary Figure 1** – Visual comparison of two extreme simulation scenarios. The scatter plots display the linear relationship between simulated expression levels of a gene pair, distinguishing between Group A (blue circles) and Group B (orange triangles). (Left) High Signal-to-Noise Ratio: An ideal scenario characterised by a large slope difference ($\beta_3$=1), low residual noise (SD=0.5), and minimal outlier contamination (10%). The differential co-expression pattern is visually distinct, resulting in a highly significant interaction p value ($p=3.9\times10^{-15}$). (Right) Low Signal-to-Noise Ratio: A challenging "limit" scenario with a subtle slope difference ($\beta_3$ =0.5), high residual noise (SD=1.2), and significant outlier presence (30%). In this condition, the group-specific trends are obscured by data dispersion, resulting in a borderline p value (p=0.05). Shaded areas indicate 95% confidence intervals.

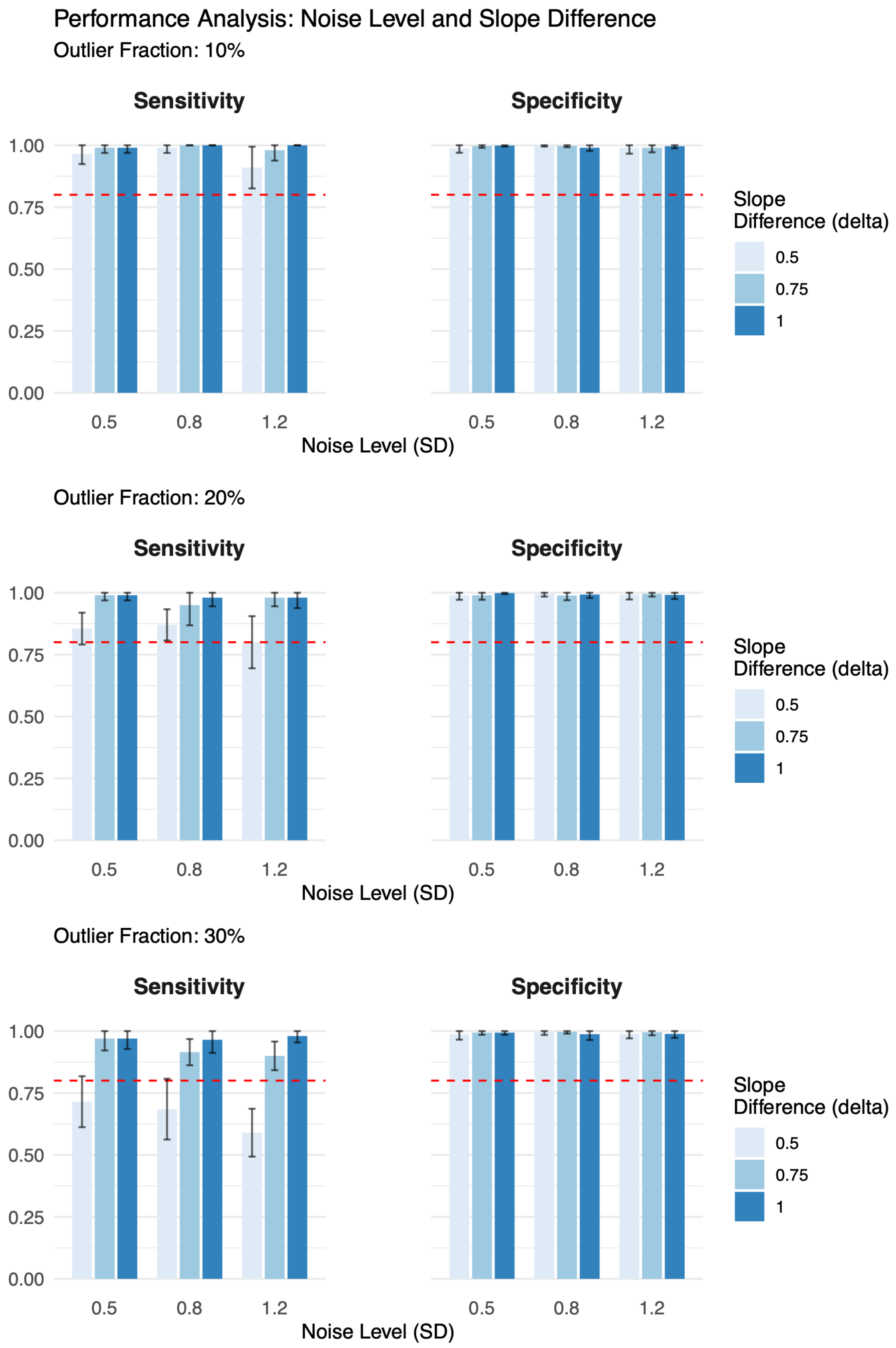


**Supplementary Figure 2** – Benchmarking of multiDEGGs performance on synthetic datasets. The bar charts illustrate the Mean Sensitivity (left panels) and Specificity (right panels) of the interaction test across 10 independent Monte Carlo simulations per parameter combination. The analysis evaluates performance variations based on: i. Outlier Fraction (panels are stratified by the percentage of samples containing outliers: 10%, 20%, and 30%); ii. Noise Level (the x-axis represents the standard deviation (SD) of the residual noise added to the linear model); iii. Interaction Strength: bar colors correspond to the magnitude of the slope difference ($\beta_3$) between the two groups). Error bars represent the standard deviation across the 10 simulations. The red dashed line marks a reference performance threshold of 0.80.

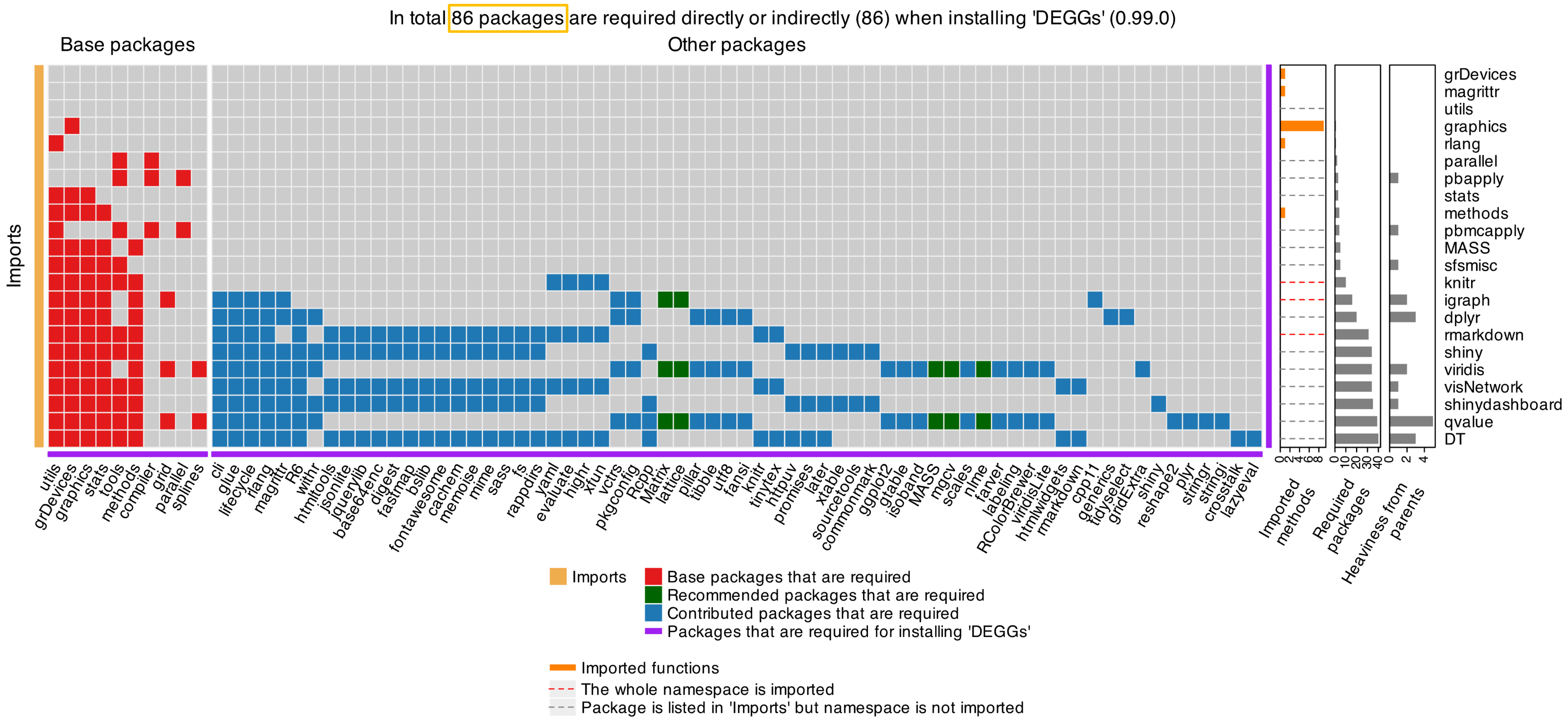


**Supplementary Figure 3** – Heatmap of package dependencies for the DEGGs package.

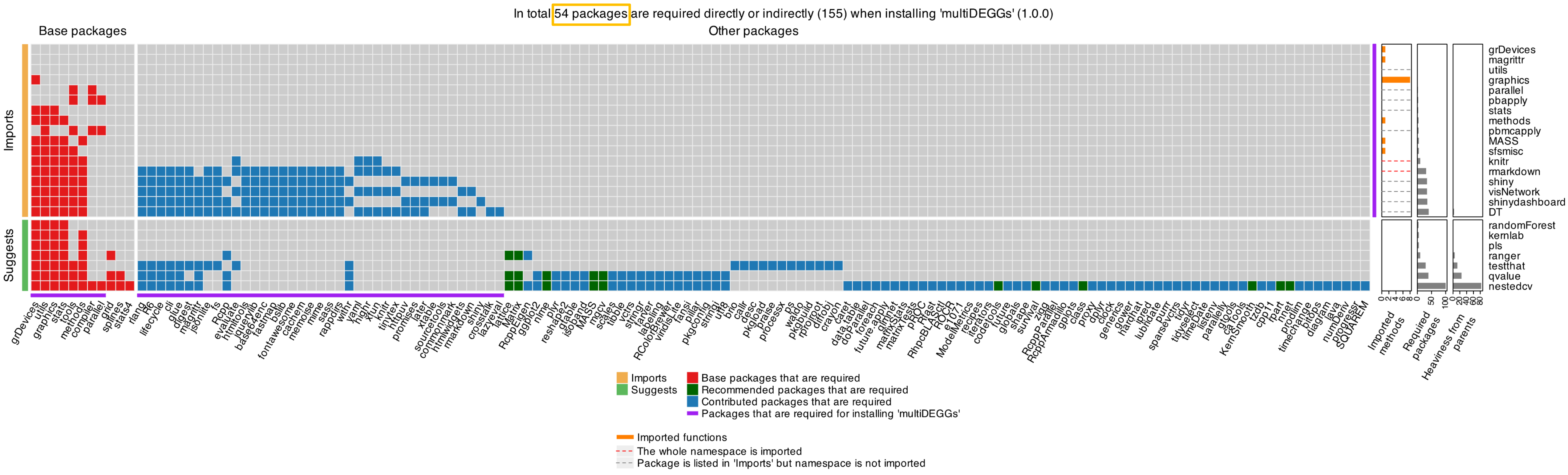

**Supplementary Figure 4** – Heatmap of package dependencies for the multiDEGGs package.

A

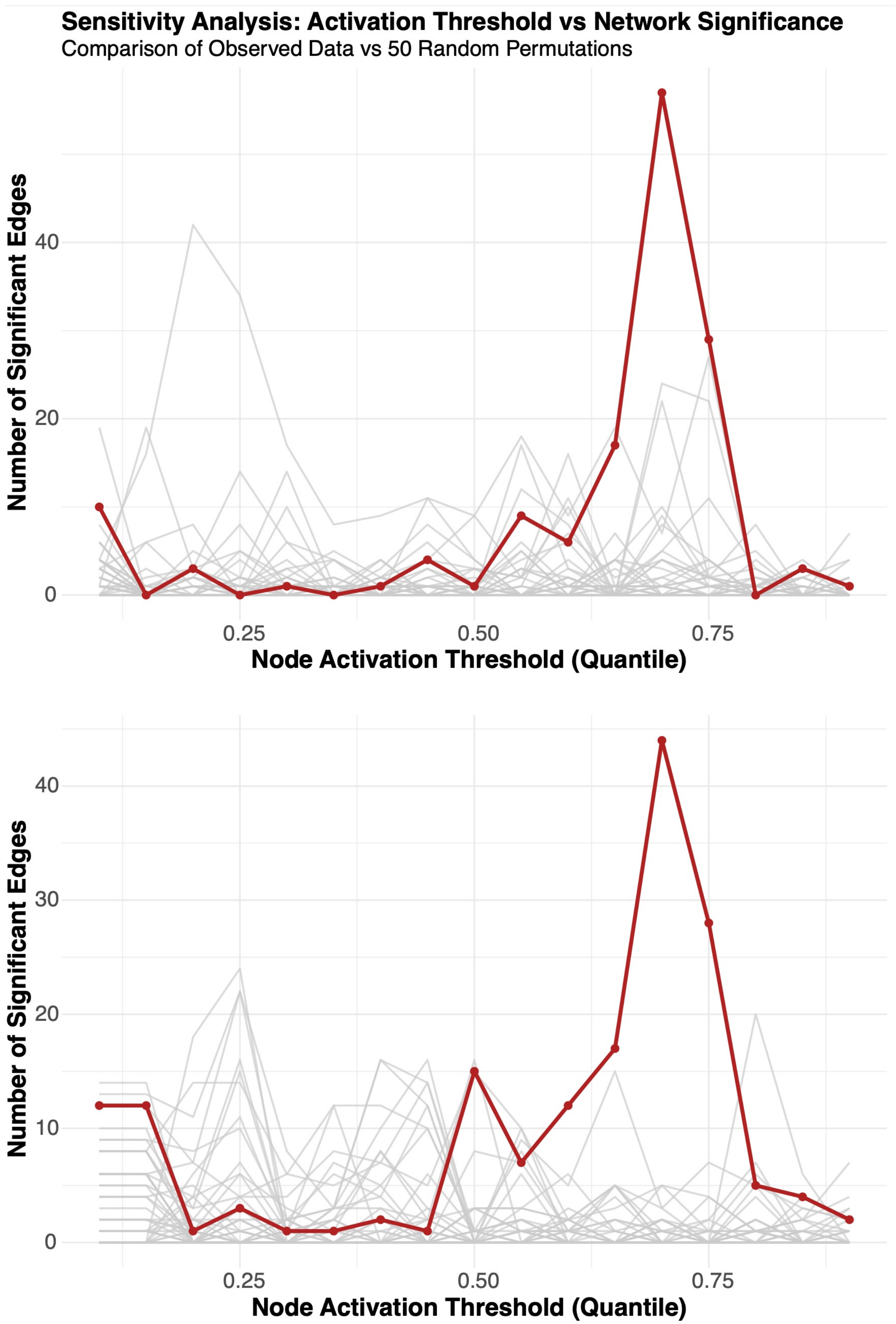


**Supplementary Figure 5** –Percolation analysis validation through permutation testing. Red lines indicate the observed number of significant edges detected at increasing node activation thresholds (from 10th to 90th percentile) in the RNA-seq data of (A) the tocilizumab cohort and (B) the rituximab cohort. Grey lines show the distribution of 50 random permutations. In both datasets, the observed biological signal peaks at the 70th percentile, demonstrating robust separation from the random background.

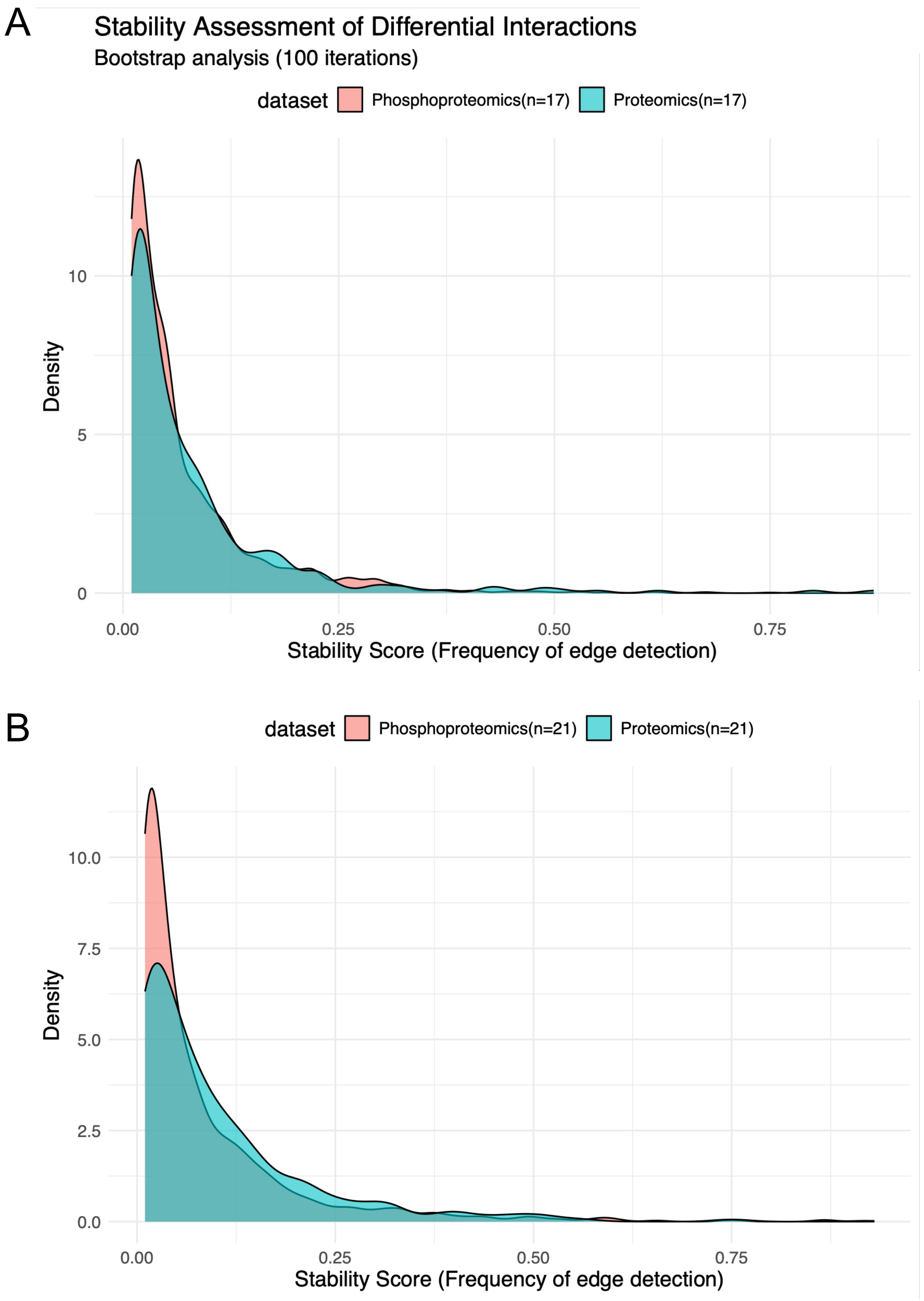


**Supplementary Figure 6 –** Bootstrap-based stability analysis for mass spectrometry proteomic and phosphoproteomic data of the Tocilizumab cohort (A) and Rituximab cohort (B). For each cohort, 100 bootstrap resamples (with replacement) were generated, and multiDEGGs was re-run to identify differential edges. The stability score represents the frequency with which each edge was detected as significant (FDR<0.05) across the 100 iterations. Density distributions show that both proteomics (blue) and phosphoproteomics (red) layers exhibit distributions heavily skewed toward low stability scores, indicating high sensitivity to sample composition. This reflects the limited sample sizes (n=17 for Tocilizumab, n=21 for Rituximab) and suggests that a substantial proportion of the identified differential interactions in these layers may not be robust.

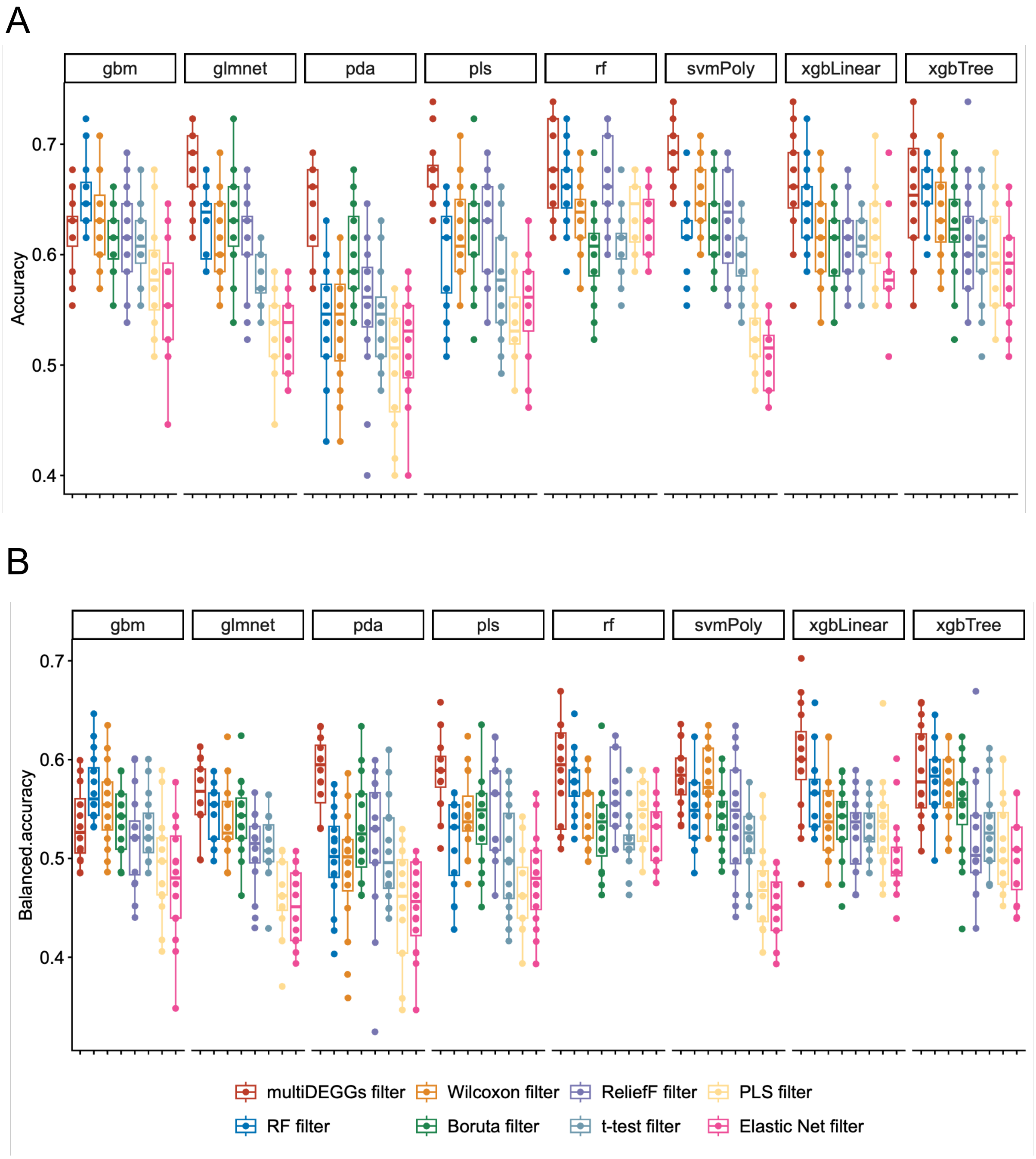


**Supplementary Figure 7** – Boxplots showing accuracy (A) and balanced accuracy (B) values of each trained model in the prediction of the tocilizumab resistant state of rheumatoid arthritis patients. Eight different models were tested: Gradient Boosting Machine (gbm), Generalized Linear Model with Elastic Net Regularization (glmnet), Penalized Discriminant Analysis (pda), Partial Least Squares (pls), Random Forest (rf), Support Vector Machine with Polynomial Kernel (svmPoly), Extreme Gradient Boosting with Linear Booster (xgbLinear), and Extreme Gradient Boosting with Tree Booster (xgbTree). For each model, multiDEGGs was sistematically compared against seven filtering methods: Wilcoxon rank-sum test, ReliefF, partial least squares (PLS), random forest, Boruta, t-test and Generalized Linear Model with Elastic Net Regularization. The maximum number of selected features was set to 40 for all filters. Each boxplot represents 16 independent repeats (individual dots) with different random splits.

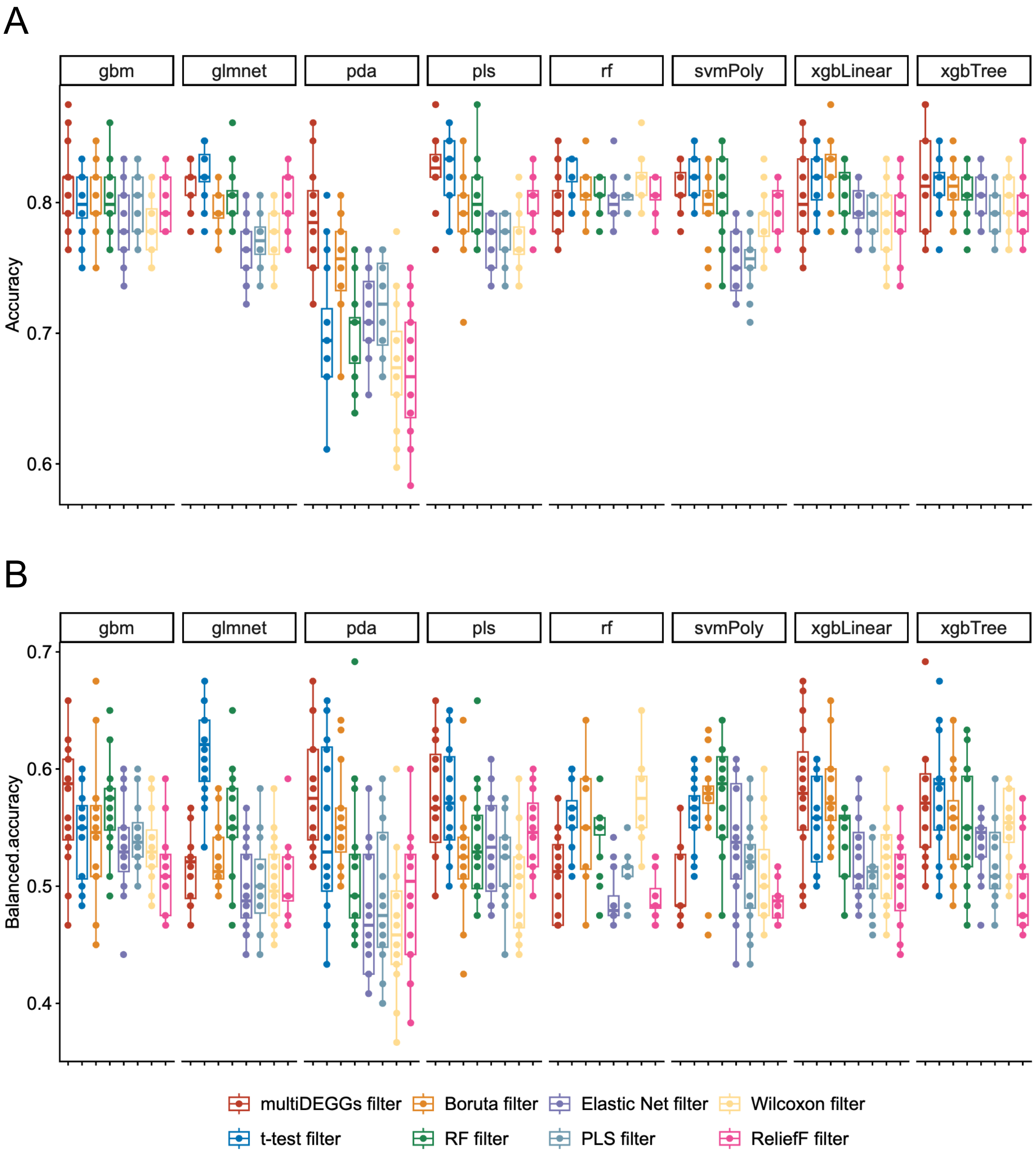


**Supplementary Figure 8** – Boxplots showing accuracy (A) and balanced accuracy (B) values of each trained model in the prediction of the rituximab resistant state of rheumatoid arthritis patients. Eight different models were tested: Gradient Boosting Machine (gbm), Generalized Linear Model with Elastic Net Regularization (glmnet), Penalized Discriminant Analysis (pda), Partial Least Squares (pls), Random Forest (rf), Support Vector Machine with Polynomial Kernel (svmPoly), Extreme Gradient Boosting with Linear Booster (xgbLinear), and Extreme Gradient Boosting with Tree Booster (xgbTree). For each model, multiDEGGs was sistematically compared against seven filtering methods: Wilcoxon rank-sum test, ReliefF, partial least squares (PLS), random forest, Boruta, t-test and Generalized Linear Model with Elastic Net Regularization. The maximum number of selected features was set to 50 for all filters. Each boxplot represents 16 independent repeats (individual dots) with different random splits.

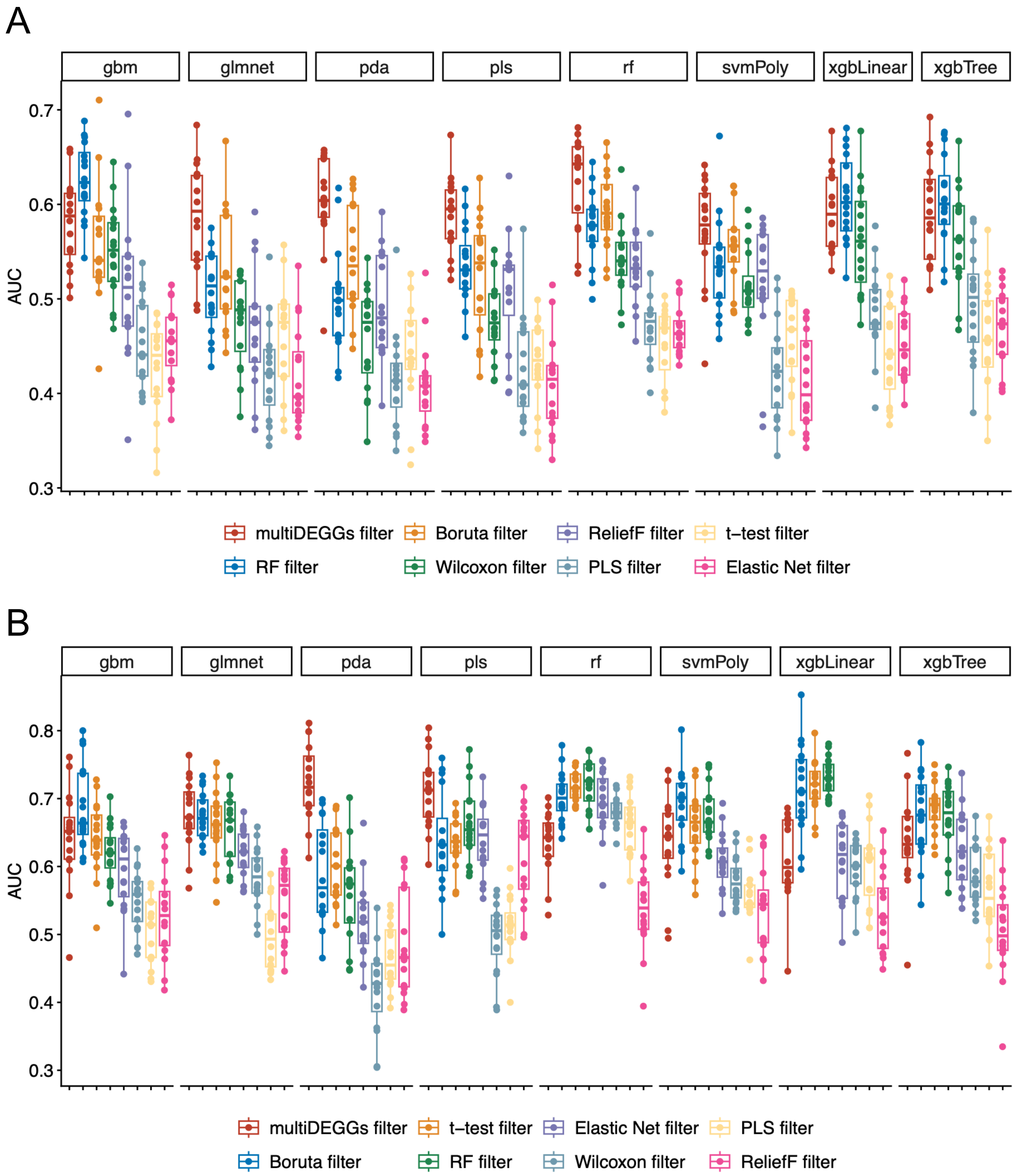


**Supplementary Figure 9** – Boxplots showing AUC values of each trained model in the prediction of the tocilizumab (A) and rituximab (B) resistant state of rheumatoid arthritis patients. Eight different models were tested: Gradient Boosting Machine (gbm), Generalized Linear Model with Elastic Net Regularization (glmnet), Penalized Discriminant Analysis (pda), Partial Least Squares (pls), Random Forest (rf), Support Vector Machine with Polynomial Kernel (svmPoly), Extreme Gradient Boosting with Linear Booster (xgbLinear), and Extreme Gradient Boosting with Tree Booster (xgbTree). For each model, multiDEGGs was systematically compared against seven filtering methods: Wilcoxon rank-sum test, ReliefF, partial least squares (PLS), random forest, Boruta, t-test and Generalized Linear Model with Elastic Net Regularization. The maximum number of selected features was set to 50 for all filters. The maximum number of selected features was set to 15 for tocilizumab (A) and to 25 for the larger rituximab cohort (B) for all filters, with multiDEGGs features restricted to combined features only. Each boxplot represents 16 independent repeats (individual dots) with different random splits.
