# Supplementary material for: multiDEGGs: Single or Multiomic Differential Network Analysis for Biomarker Discovery and Feature Engineering for Predictive Modeling
Source: Comput Struct Biotechnol J. 2026 Mar 18;35(1):0001. doi: 10.34133/csbj.0001 (PMC13082464; doi:10.34133/csbj.0001)
Supplement: Supplementary 1 — Supplementary Methods Figs. S1 to S9 Files S1 to S3 Movie S1 [file csbj.0001.f1.zip › Supplementary Methods.docx]

**SUPPLEMENTARY METHODS**

**Benchmarking of multiDEGGs with simulated data**

To assess the performance of multiDEGGs in detecting differential co-expression patterns, specifically its sensitivity to the interaction term ($\beta_{3}$) and robustness against noise and outliers, we performed a comprehensive benchmarking analysis using simulated RNA-seq data.

We generated synthetic datasets for a cohort of patients, randomly assigned to two biological groups (Group A and Group B) with balanced probability ($p=0.5$). For each simulation, we constructed a reference network of disjoint gene pairs. To mimic a realistic biological scenario where true differential interactions are sparse, we imposed a ground truth of only 20% of true differential pairs (True Positives, TP), while the remaining 80% were stable across groups (True Negatives, TN).

Co-expressions of genes were simulated based on a linear relationship with injected interaction effects. For a given gene pair (*u,v*), the expression of the target gene was modelled as:

$$u_{i}=\beta_{0}+\beta_{1}v_{i}+\beta_{2}k_{i}+\beta_{3}\left( v_{i}\cdot k_{i} \right)+\varepsilon_{i}$$

where:

- $k_{i}$ is the binary group indicator (0 for Group A, 1 for Group B).
- $\beta_{1}$ represents the baseline slope (correlation) in the reference group.
- $\beta_{2}$ represents the group-specific shift in expression (intercept difference), implemented as fixed shifts in the simulation.
- $\beta_{3}$ is the interaction coefficient, representing the true difference in slopes between groups. In our simulation code, this corresponds to the parameter delta_beta ($\left| \beta_{A}-\beta_{B} \right|$.).
- $\varepsilon_{i}$ represents Gaussian noise with standard deviation $\sigma_{noise}$.

We evaluated the method's performance across a grid of parameters representing varying degrees of data quality and signal strength:

1. **Noise Level** ($\sigma_{noise}$): we tested low, medium, and high dispersion of residuals relative to the regression line ($\sigma\in\{0.5, 0.8, 1.2\}$).
2. **Interaction Strength** ($\beta_{3}$): we varied the difference in slopes between groups from subtle to strong effects ($\beta_{3} \in\{0.5, 0.75, 1.0\}$).
3. **Outlier Fraction**: to test the robustness of the regression, we introduced random uniform noise (deviating significantly from the linear trend) to a subset of samples ({10%, 20%, 30%} of the total cohort).

To illustrate the range of conditions tested, Supplementary Figure S1 displays representative regression plots for two extreme simulation scenarios: a strong-signal case (ideal conditions) and a weak-signal case (characterised by high noise and outlier contamination).

For each combination of parameters, we ran 10 independent Monte Carlo simulations. Performance was evaluated using Sensitivity (Recall) and Specificity at a nominal significance threshold of $p<0.05$*.*

The results of the benchmarking, are presented in Supplementary Figure S2 and fully detailed in Supplementary File 1.

Across all 27 tested conditions, the method demonstrated good specificity, consistently exceeding 99% (mean specificity: 0.993 ± 0.013). Sensitivity was highly dependent on the signal-to-noise ratio. In conditions with low noise (SD = 0.5) and minimal outliers (10%), the method achieved a mean sensitivity of 96.5% for moderate signals (β_3_ = 0.5) and 100% for strong signals (β_3_ = 1). As expected, sensitivity decreased in more challenging scenarios. For weak interaction signals (β_3_ = 0.5), increasing the noise level to and outlier fraction resulted in a sensitivity drop to 59.0%. However, for stronger signals (β_3_ ≥ 0.75), the method remained robust, maintaining a sensitivity above 88% even in the worst-case noise/outlier conditions.

The observed reduction in sensitivity in high-noise/high-outlier scenarios aligns with the visual inspection of the data. As illustrated in Supplementary Figure S1 (right panel), gene pairs characterised by high residual dispersion (SD = 1.2) and substantial contamination (30% outliers) exhibit overlapping distributions that obscure the interaction effect. In these 'limit' scenarios, multiDEGGs exhibits a conservative behaviour, avoiding potential over-interpretation of noisy data.

# Stability analysis for low-sample datasets

While multiDEGGs provides a flexible framework for multi-omic integration, the reliability of the inferred differential networks is intrinsically dependent on the quality and statistical power of the input data. In our case studies, the proteomics and phosphoproteomics layers had limited sample sizes (n=17 for the Tocilizumab cohort and n=21 for the Rituximab cohort). These data were included primarily to demonstrate the package's functionality in handling multi-layer structures and bridging diverse omic data types.

To quantify the impact of sample size on network robustness, we performed a stability analysis using bootstrapping. We generated 100 bootstrap resamples (with replacement) for each dataset and re-ran multiDEGGs to calculate the stability score, defined as the frequency with which each edge was detected as significant across iterations.

The results, presented in Supplementary Figure 4, show a density distribution heavily skewed towards low stability scores for the proteomic and phosphoproteomic layers. This indicates that a substantial portion of the differential interactions identified in these specific layers may be sensitive to sample composition and should be interpreted with caution. We strongly recommend that users apply multiDEGGs to datasets with adequate sample sizes relative to the number of features and biological variability, or perform similar stability assessments to validate the robustness of their findings.

**Rheumatoid arthritis cohorts**

multiDEGGs was applied to data collected from two rheumatoid arthritis (RA) clinical trials whose details are provided in the following sections.

R4RA (Tocilizumab arm)

The *Randomised, open labelled study in anti-TNFa inadequate responders to investigate the mechanisms for Response - Resistance to Rituximab versus Tocilizumab in RA (R4RA)* includes patients with difficult-to-treat RA, who had previously failed both conventional DMARD therapy and at least one biologic agent (anti-TNF). This clinical trial is described in full in (Rivellese et al. 2022) and the RNA-seq data used in this paper is available in ArrayExpress with accession number [E-MTAB-11611](https://www.ebi.ac.uk/biostudies/ArrayExpress/studies/E-MTAB-11611?query=E-MTAB-11611%20)(Cubuk and Lewis 2022).

In brief, transcripts were quantified using Salmon46 v.0.13.1 and an index generated from the Gencode release29 transcriptome following the standard operating procedure. Tximport v.1.13.10 was used to aggregate transcript-level expression data to genes, then counts were subjected to variance-stabilizing transformation (VST) using the DESeq2 v.1.25.9 package. Details of sequencing and library preparation are described in (Rivellese et al. 2022).

Mass spectrometry-based proteomics and phosphoproteomics profiles were generated on synovial biopsies from R4RA as described in (Çubuk et al. 2024). Both matrices underwent quantile normalisation and log2 transformation.

Plasma O-link was performed on baseline blood samples (combined panel size = 732). The normalised protein expressions were obtained from a Sanofi panel with 700 analytes.

STRAP (Rituximab arm)

The *Stratification of Biologic Therapies for RA by Pathobiology (STRAP)* includes patients treated with rituximab who had failed DMARD therapy but were biologic-naïve. The clinical trial is described in full in (Lewis et al. 2025) and the RNA-seq data used in this paper is available in ArrayExpress with accession number [E-MTAB-13733](https://www.ebi.ac.uk/biostudies/ArrayExpress/studies/E-MTAB-13733?query=E-MTAB-13733) (Cubuk and Lewis 2025).

Transcripts were quantified using Salmon version 0.13.153. Tximport version 1.13.10 was used to aggregate transcript-level expression data to genes, then counts were subjected to variance-stabilising transformation (VST) using the DESeq2 version 1.25.9 package. Details of sequencing and library preparation are available in (Lewis et al. 2025).

Mass spectrometry-based proteomics and phosphoproteomics results were generated on synovial biopsies from STRAP as described in (Çubuk et al. 2024). Both matrices underwent quantile normalisation and log2 transformation.

**Parameter configuration for feature selection methods**

The table below summarises the parameters used for each of the eight feature selection methods employed for treatment response prediction in the two rheumatoid arthritis cohorts analysed in this work.

| Filter | Parameter Tuning | Number of features  (Tocilizumab Cohort) | Number of features  (Rituximab Cohort) |
| --- | --- | --- | --- |
| multiDEGGs filter | - | automatic, max 40 | automatic, max 50 |
| RF filter | ntree = 1000 | 40 | 50 |
| Wilcoxon filter | - | 40 | 50 |
| Boruta filter | maxRuns=100, pValue=0.01 | automatic, max 40 | automatic, max 50 |
| ReliefF filter | - | 40 | 50 |
| t-test filter | - | 40 | 50 |
| PLS filter | - | 40 | 50 |
| Elastic Net filter | α ⊂ (0.7 ,7) λ = min cross-validation error | automatic, max 40 | automatic, max 50 |

**References**

Çubuk, Cankut, et al. (2024), ‘Phosphoproteomic Profiling of Early Rheumatoid Arthritis Synovium Reveals Active Signalling Pathways and Differentiates Inflammatory Pathotypes’, *Arthritis Research and Therapy*, 26/1, https://doi.org/10.1186/s13075-024-03351-4.

Cubuk, Cankut, and Myles Lewis (2022), ‘RNA-Seq of Synovial Tissue Biopsies from Individuals with Rheumatoid Arthritis from the R4RA Randomised Clinical Trial’, *E-MTAB-11611 ArrayExpress, Biostudies*.

Cubuk, Cankut, and Myles Lewis (2025), ‘RNA-Seq of Synovial Tissue Biopsies from Individuals with Rheumatoid Arthritis from the STRAP Randomised Clinical Trial’, *E-MTAB-13733 ArrayExpress, Biostudies*.

Lewis, Myles J., et al. (2025), ‘Deep Molecular Profiling of Synovial Biopsies in the STRAP Trial Identifies Signatures Predictive of Treatment Response to Biologic Therapies in Rheumatoid Arthritis’, *Nature Communications*, 16/1: 5374, https://doi.org/10.1038/s41467-025-60987-9.

Rivellese, Felice, et al. (2022), ‘Rituximab versus Tocilizumab in Rheumatoid Arthritis: Synovial Biopsy-Based Biomarker Analysis of the Phase 4 R4RA Randomized Trial’, *Nature Medicine*, 28/6: 1256–68.
