# Supplementary figures and images for: multiDEGGs: Single or Multiomic Differential Network Analysis for Biomarker Discovery and Feature Engineering for Predictive Modeling
Source: Comput Struct Biotechnol J. 2026 Mar 18;35(1):0001. doi: 10.34133/csbj.0001 (PMC13082464; doi:10.34133/csbj.0001)

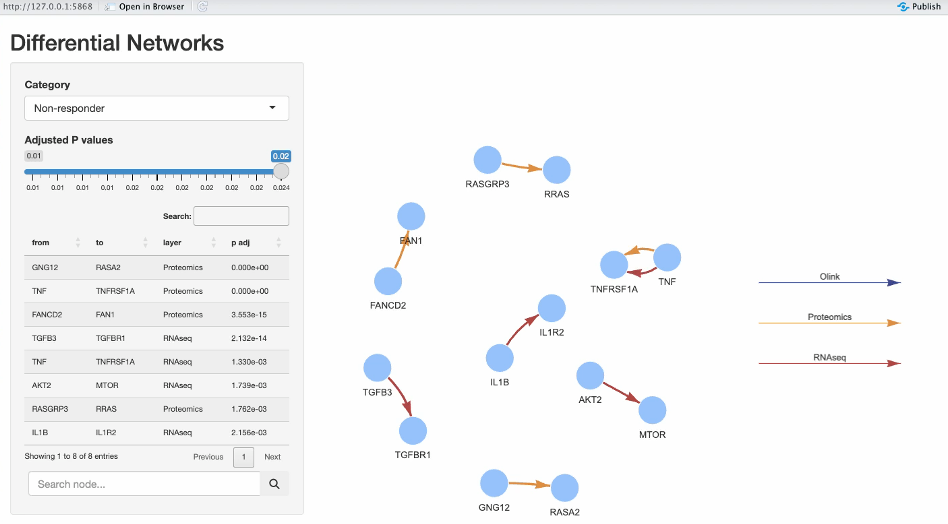

Supplement: Supplementary 1 — Supplementary Methods Figs. S1 to S9 Files S1 to S3 Movie S1 [file csbj.0001.f1.zip › Video1.png]
